# Supplementary material for: Evaluation of Prognostic Factors of Severity in Acute Biliary Pancreatitis
Source: Int J Mol Sci. 2020 Jun 16;21(12):4300. doi: 10.3390/ijms21124300 (PMC7352282; doi:10.3390/ijms21124300)
Supplement: Supplementary file 1 [file ijms-21-04300-s001.pdf]

Table S1: Demographic and clinical characteristics of the five patients who died of severe acute biliary pancreatitis.

Patient	Age	Gender	BMI	Charlson Index	Hospital stay	time	WBC	NLR	PLR	SIRI	CRP	PCT	Hepcidin	BISAP	SIRS	MMS
1	89	male	24.4	4	24	admission	22.03	25.78	210.39	4.87	417.1	9.94	158.6	2	1	3
						48 h	17.78	24.28	199.18	8.98	415.5	9.23	231.1	5	2	4
2	68	male	22.9	2	3	admission	22.13	8.73	127.31	5.66	5.0	0.49	284.6	1	1	2
						48 h	3.96	6.43	360.87	1.09	446.8	16.28	161.1	5	4	4
3	92	female	21.2	4	3	admission	25.27	15.43	210.88	11.57	270.1	33.45	134.6	4	3	4
						48 h	14.54	23.18	464.91	12.05	565.7	26.11	311.0	5	3	4
4	93	male	28.3	4	10	admission	17.57	37.81	453.49	23.44	36.1	0.60	12.0	3	2	0
						48 h	17.51	29.94	400.00	18.57	256.9	0.57	330.0	5	2	1
5	82	female	23.0	4	34	admission	15.12	17.25	437.97	9.32	10.3	0.56	246.5	3	2	1
						48 h	19.00	15.47	245.05	8.20	337.9	3.09	411.4	4	3	2

BMI: body mass index; WBC: white blood count; NLR: neutrophil-lymphocyte ratio; PLR: platelet-lymphocyte ratio; SIRI: Systemic Inflammatory Response Index ; CRP: C-reactive protein ; PCT: procalcitonin; BISAP: Bedside index for severity in acute pancreatitis; SIRS: Systemic Inflammatory Response Syndrome ; MMS: Marshall modified score
